# Childhood Disintegrative Disorder (CDD): Symptomatology of the Norwegian Patient Population and Parents’ Experiences of Patient Regression

**DOI:** 10.1007/s10803-021-05023-7

**Published:** 2021-05-02

**Authors:** Martin John Ellis, Kenneth Larsen, Sophie Seychelle Havighurst

**Affiliations:** 1 University of Oslo, Oslo, Norway; 2 Oslo University Hospital, Oslo, Norway; 3 University of Melbourne, Melbourne, Australia

**Keywords:** CDD, Childhood Disintegrative Disorder, Heller Syndrome, Autism, Regression

## Abstract

Childhood Disintegrative Disorder (CDD) is a rare and little researched developmental disorder characterised by regression in language and social skills after a period of seemingly normal development until at least the age of 2 years. The study contacted all parents of CDD patients in Norway to assess patient symptomatology and parents’ experiences of regression via questionnaire or interview. There were 12 participants. Symptomatology was in-line with previous studies, with universal regression in language and social skills and onset predominantly at 2–4 years. Regression was connected to feelings of ‘loss’ and uncertainty over the prognosis for CDD patients. The study supported CDD diagnostic criteria and showed that CDD patient regression has profound implications for parental well-being.

Childhood Disintegrative Disorder (CDD) is a rare and little understood form of pervasive developmental disorder (PDD). CDD is typified by a period of normal development in the child for at least two years before a period of profound and irreversible regression in social and cognitive skills (Mehra et al., 2018). After regression, behavioural traits in CDD patients are similar to autism. However, the seemingly normal development of the CDD patient before regression onset, as well as the late age of regression onset, are typically considered specific to the condition and markers of its distinctness from other autism spectrum disorder (ASD) diagnoses (Mehra et al., 2018; Siperstein & Volkmar, 2004; Volkmar, 1992). The only systematic review of research on CDD (Mehra et al., 2018) concluded that prevalence was between 1.1 and 9.2 per 100,000, suggesting CDD is between 32 and 283 times rarer than ASD. The rarity of CDD has made research on the condition extremely challenging and uncertainty regarding its diagnostic characteristics, and even its diagnostic validity, remain.

CDD was first described by Theodor Heller in 1908 and termed ‘dementia infantilis’ (Mouridsen, 2003). Since this time a variety of terms have been used for what, to all intents and purposes, seem to be the same diagnosis. The ICD-10 includes under the category ‘other childhood disintegrative disorder’ (F84.3): ‘dementia infantilis’, ‘disintegrative psychosis’, ‘Heller syndrome’, and ‘symbiotic psychosis’. In practice, all of these terms (except ‘symbiotic psychosis’, which is almost unheard of in the literature) are used interchangeably (Kurita et al., 2005). More commonly, the corresponding DSM-IV term ‘Childhood Disintegrative Disorder’ (CDD) is seen in the literature, and is again used interchangeably (Mouridsen, 2003; Volkmar & Rutter, 1994).

Despite the evidence for the distinctive validity of CDD, the term CDD has been incorporated into the diagnostic category of ‘ASD’ in the DSM-V. To date, the corresponding term ‘other childhood disintegrative disorder’ (F84.3), is still employed in the ICD-10. Indications are that the ICD-11 will follow the DSM-V in incorporating CDD into a broader ASD category (World Health Organization, 2019). As Mehra et al. (2018) highlight, failing to include a diagnostic ‘marker’ for regression, or global loss of previously acquired skills, will make future identification and research on this patient group even more challenging. From the limited information available it seems the ICD-11 may take this into account and include a separate marker for regression of skills (Autism Europe, 2018).

Following the concerns in Mehra et al. (2018) about diagnostic classification changes potentially making it more challenging to identify patients with late-onset regression, and in light of the significant amount of research suggesting that the distinctive symptomatology of patient cases with late onset regression argues persuasively for CDD constituting a distinct diagnosis (Pelphrey et al., 2012; Mehra et al., 2018; Mouridsen, 2003; Kurita et al., 2005; Volkmar & Rutter, 1994; Bray et al., 2002), this study maintains usage of the term CDD for the patient group.

Whilst regression in social and cognitive skills is reported in studies of patients with autism (Barger et al., 2013) there has generally been a consensus within the field of autism research that CDD constitutes a diagnosis distinct from ‘autism with regression’. Both the late age of regression onset and the domain-general nature of regression in CDD are cited as significant factors indicating differences from regression otherwise observed in patients with autism (Kurita et al., 2005; Mehra et al., 2018; Mouridsen, 2003; Volkmar & Rutter, 1994). Mehra et al. (2018) assessed the regression ages in CDD patients from all available research (combined total: n = 70) and found a mean regression age of 3 years 2 months (range between 2 and 7 years). This represents a significantly later age of regression than in autism cases (Meilleur & Fombonne, 2009; Werner et al., 2005) and suggests a high likelihood of differing aetiology between the two conditions. Studies also tend to indicate no previous signs of atypical development in CDD patients prior to the beginning of regression symptoms, whilst atypical development seems to be more usual in autism (Mehra et al., 2018; Siperstein & Volkmar, 2004; Volkmar, 1992). Mehra et al. (2018) found that across four epidemiological studies, 100% of CDD patients (n = 29) had reached all age-appropriate developmental milestones before the onset of symptoms. In contrast, only 10% of control ASD cases assessed (n = 21) and 14% of ASD ‘with regression’ cases (n = 51) had reached all age appropriate developmental milestones prior to regression.

Diagnostic differentiation studies of CDD have highlighted other possible factors in ascertaining the clinical distinctiveness of the condition. These factors include: higher rates of eye-contact in CDD subjects (Gupta et al. 2017); significantly greater fearfulness, or even hallucinations, in the child at onset (Agarwal et al., 2005; Kurita, Koyama, et al., 2004; Westphal et al., 2013); higher rates of epilepsy (Kurita, Koyama, et al., 2004; Kurita, Osada, et al., 2004; Rosman & Bergia, 2013); more general loss of adaptive skills such as bowel and bladder control (Mehra et al., 2018); and higher rates of mutism (Bray et al. 2002; Volkmar & Rutter, 1994). Comorbid features associated with CDD are sleep problems (Mehra et al., 2018), challenging behaviour (Malhotra & Gupta, 2002; Mehra et al., 2018), and self-harm (Mehra et al., 2018).

Regarding the speed of onset of regression in CDD, studies sometimes divide between ‘acute’ onset (in only days or weeks) and ‘insidious’ onset (over weeks or months), though there is little consensus as to whether either is more common (Malhotra & Gupta, 2002; Volkmar & Cohen, 1989). Some suggest that the speed of this regression may also indicate differences between CDD and ASD (Mouridsen et al., 1999). Several studies have suggested poorer long-term outcomes for CDD patients than for patients with ASD diagnoses, though there is little indication as to why this may be (Matson & Mahan, 2009; Mouridsen et al., 1999).

To date, there has been very little research documenting the process of regression in CDD patients (an exception to this is a case study by Palomo et al. 2008, which employs home videos and clearly shows the patient’s marked decline in spontaneous language, social gaze, and development of repetitive behaviour). More detailed reporting on symptom onset, including timeframe and chronology, would help inform research on the disorder. There is also a lack of research regarding how regression affects the patient’s family.

The lack of research regarding regression onset in CDD, as well as the longer-term outcomes of the disorder, may have hindered the establishment of the diagnostic validity of the condition. This may also have contributed to non-uniform application of diagnostic terminology. A study by Volkmar and Rutter (1994) assessed a sample of 977 people with a ‘developmental disturbance diagnosis’ (autism, ‘autistic-like’ conditions, non-PDD disorders) and found that 16 patients had been given a diagnosis of CDD. A further 10 patients met criteria to be given a CDD diagnosis but had not been given this diagnosis by their clinician, and seven cases fell ‘just short’, primarily because of uncertainty regarding the age at which regression occurred. There may also be some overlap between the diagnosis of CDD and the diagnostic category Pervasive Developmental Disorder—Not Otherwise Specified (PDDNOS) (Kurita et al., 2005). (Once again PDDNOS as a diagnosis is now incorporated into the ‘ASD’ category under the DSM-V.)

A limited amount of research has assessed the potential causes of CDD. Gupta et al. (2017) found several rare variant candidate genes in CDD patients (although the study could not determine that any of these were ‘clearly deleterious’). Specifically, Gupta et al. (2017) point to several candidate genes which are potentially involved in transcription (TRRAP, ZNF236, and KIAA2018) as transcription involvement is a characteristic of many genes currently considered as potentially associated with ASD. Furthermore, the brain expression profile of these genes resembled the expression profile of variant genes in ASD subjects ‘with regression’ (but not subjects with autism without regression). Of particular note is that these genes are likely to be most associated with expression in non-neocortical regions between the ages of 1 to 12 years (Gupta et al., 2017). Philippe et al. (2015) also suggest potential genetic causes. This would seem to further support the argument that there is merit in defining ASD/CDD patients whose development regresses separately. Further case studies have proposed connections between CDD and Vitamin B12 deficiency (Malhotra et al., 2013) and with glycoprotein sialylation deficiency (Barone et al., 2016). Finally, due to the occasionally sudden nature of onset in CDD, and the high prevalence of epilepsy among CDD patients, some have suggested links to Landau-Kleffner syndrome (a rare form of epileptiform aphasia) (Deonna & Roulet-Perez, 2010; Stefanatos et al., 2002). However, the majority of CDD patients show no abnormalities on electroencephalography (EEG) readings (Mehra et al., 2018), and some CDD patients do not have epilepsy, so the two groups remain distinct.

Whilst there are good arguments for ‘tidying’ the application of the terminology of ASD, CDD, and PDDNOS diagnoses and incorporating them into a broader ASD diagnosis, it can also be argued that ‘late onset’ regression cases (whether or not these are termed ‘CDD’) require further clarification. Given the significant likelihood of CDD patients (potentially alongside other patients with ‘late onset regression’) constituting an aetiologically distinct group, it would seem pertinent to further focus on these cases specifically. Research on both the symptom picture of CDD patients and the specific nature of the regression process itself would help resolve some of these issues.

As CDD is such a rare condition and obtaining direct access to medical files for research is problematic, this study employed the Norwegian Patient Registry to identify all patients with the diagnosis in Norway. Parents/guardians from this national register were contacted via treatment services and invited to take part in the study which collated information regarding symptom onset and regression. The aims of the study were to assess: (1) Parent/guardian reports of CDD patient symptomatology; (2) Parents/guardians of CDD patients’ reports of the regression phase of the CDD patient and the effects, both practically and emotionally, this had had on the family.

## Method

### Participants

The inclusion criterion was being the parent or guardian to a patient diagnosed under ICD-10: F84.3 ‘other disintegrative disorder in childhood’ in Norway. There were 12 participants in the study, and all were the biological parent of a patient with CDD according to NPR (‘Norwegian Patient Registry’). In ten cases the primary respondent was the mother and in two cases the father. Additionally, in three questionnaire responses the participant indicated that both parents of the patient had responded together, and in one case the mother reported responding together with a sibling of the patient. All participants responded in Norwegian.

### Procedure

**Fig. 1:**
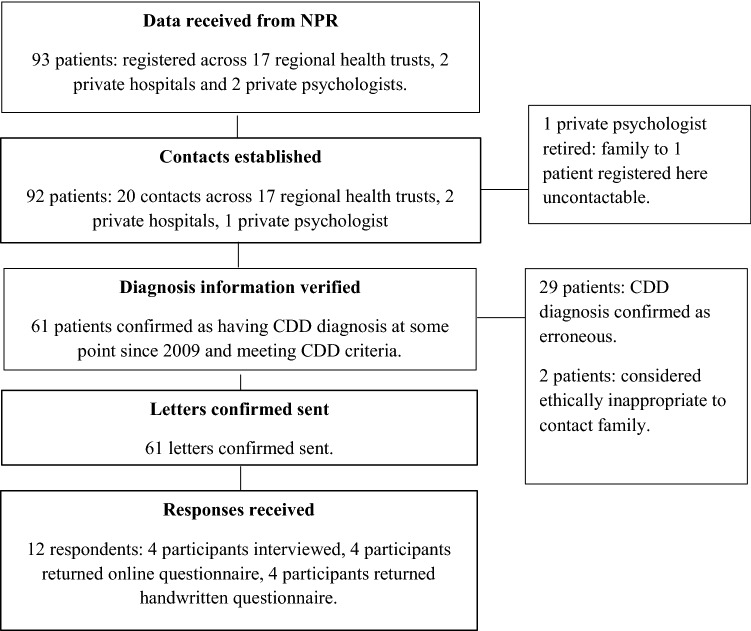
Participant flow diagram

The study was part of a larger project carried out at the University of Oslo in collaboration with Oslo University Hospital. The project was approved by the Regional Committee for Medical and Health Research Ethics Regional Committee for Medical and Health Research Ethics, South-East and NPR. The project aimed to contact all parents/guardians of patients with a CDD diagnosis in Norway. Participants were identified indirectly in a ‘two-stage’ process via relevant treatment service personnel (see below and Fig. 1). Letters informing of the study, consent forms, and questionnaires were sent by post to the patients’ families via a contact at the treatment service where the patient was registered. All participation was voluntary, and if the family decided not to respond to the invitation, their complete anonymity to the research team was thus maintained.

Stage 1 entailed establishing a ‘contact’ at each treatment service where there existed CDD patients according to NPR data. To enable this, NPR provided the first author with an overview of the last registered treatment service for each of the 93 registered CDD patients in Norway. Contacts were required to be health personnel with access to the patient journal system and were established for all but one of the 21 listed hospitals/psychologists, encompassing 92 patients. Contacts’ details were forwarded securely by the author to NPR. Contacts were also sent letters for forwarding to the families of patients by post.

Stage 2 of the process comprised all treatment service contacts receiving an encrypted email of patient-identifying information from NPR. The contacts used this information to ascertain the patient’s family’s address from the journal system and forward the family the invitation to take part in the study. Before sending invitations, patient diagnosis information was validated by the treatment service personnel. This showed that a significant number of patients (n = 29) did not in-fact have a diagnosis of CDD (these cases could represent, for example, patients who had been assessed for the condition at some point, but were confirmed as having another diagnosis, or simple code registration errors. However, to safeguard anonymity, specific information as to the reason for excluding patients was not forwarded to the researchers). Treatment service personnel also assessed the appropriateness of sending out invitations, and a further two families were not contacted because of ethical considerations (specific information regarding the nature of these ethical reasons was not transmitted to the research team). A total potential sample of 61 patients were identified in Norway, and their families were sent letters inviting them to take part.

Participants were given the option of taking part either by returning the printed questionnaire in the pre-paid envelope provided or contacting the research team by email if their preference was to take part via online questionnaire or interview. All participation was dependent upon the return of a signed consent form, either by post, or by email. The final sample (n = 12) amounted to a return rate of just under 20%. Four respondents were interviewed (two face-to-face, two by telephone), four returned the online questionnaire, and four returned the handwritten questionnaire. All responses were in Norwegian. Interviews were recorded using a Dictaphone, transcribed to individual documents by the author, anonymised, and translated to English. Interviews lasted between 75 and 95 min. Written and online responses were transcribed, anonymised, and translated in the same manner. All data was securely stored via the Services for Sensitive Data (TSD) server at the University of Oslo.

### Measures

The lack of previous research on the families of CDD patients necessitated designing a questionnaire and interview-guide specifically for the project. The project employed quantitative scales assessing patient symptomatology and in-depth qualitative responses on parents’ experiences of CDD, their family life, and their experiences of healthcare and support services (reported separately). This study focuses on parental responses regarding CDD patient symptomatology and responses specifically regarding parents’ experiences surrounding the regression of the CDD patient.

The questionnaire comprised of 36 questions: 30 of which were designed for all participants to answer. Three additional questions were specifically for participants whose child received care services (either full-time or respite), and three additional questions were for participants with more than one child in the family (these aimed at assessing parents’ perceptions of the effects of the disorder on siblings). Of the 30 core questions: 8 focused on elements of diagnosis and symptomatology (these were primarily quantitative and followed a yes/no, or present/not present format). A further 22 questions focused on the parent’s experiences with their child’s diagnosis and support (5 of these were quantitative measures on a 1–7 scale; 17 were ‘open’ qualitative questions.) Three of the ‘open’ questions focused specifically on elements regarding the regression phase of the CDD patient. An interview guide was also developed alongside the questionnaire and mirrored the questionnaire content (whilst allowing for a more ‘responsive’ discussion of the topics). All of the quantitative measures were also asked in the interviews.

Demographic information comprised of the relation of the participant to the CDD patient, the gender of the CDD patient, the current age of the CDD patient, and whether the CDD patient lived at home with the family, received respite care, or lived in full-time residential care. Participants were then asked a yes/no question regarding whether they believed the CDD patient had shown obvious signs of regression in cognitive and social abilities. Participants were further asked to report the presence or absence of the following symptoms in the patient: loss/decline of language skills; loss/decline of social skills; loss/decline of motor skills; loss/decline of toilet training abilities; hallucinations; fear or awareness ‘something was wrong’ (in the patient at regression onset); developing repetitive behaviours. These correspond to the most commonly observed symptoms in CDD regression (Mehra et al, 2018). In addition, participants were asked to report the age of regression onset.

In addition to the three ‘open’ qualitative questions addressing the issue of regression in the CDD patient, participants were asked three questions on a 1–7 scale assessing the effects of patient regression on the family (see Table 2).

### Analysis

The quantitative responses were transferred to Excel worksheets by the first author where response frequencies were compiled and converted to percentages.

The qualitative analysis procedure followed a standard approach found in thematic analysis, based upon the six stages delineated in Braun and Clarke (2006). These stages are: (1) familiarising yourself with the data; (2) generating initial codes; (3) searching for themes; (4) reviewing themes; (5) defining and naming themes; (6) producing the report. Initially 108 codes were employed in the thematic analysis which were revised down to 18 themes. These themes were given titles based on participants’ own words, rather than employing researcher-imposed definitions. Of these 18 themes, one, entitled ‘I don’t understand that a personality can change so much’ incorporated parent responses to the regression of the patient.

In terms of reliability in this study, the questionnaire and interview-guide were designed for the study and, due to limitations of time and rarity of the condition, could not be tested in a pilot study beforehand. The scale measures used have also not been independently tested for validity (due to time restrictions). However, all measures used in the study were constructed by the authors in collaboration, applying the principle of ‘triangulation’ to reduce potential individual bias (Willig, 2013). This also applies to the thematic analysis, whereby coding was verified by all three authors independently, and author consensus was considered a control against bias and marker of reliability (Willig, 2013).

## Results

### Participant Demographic Information

All 12 respondents in the study were the biological parent of a patient with a CDD diagnosis (see ‘Participants’). Therefore, in both the results and discussion that follows the term ‘parent’ is employed for participants and ‘child’ refers to CDD patients.

### Quantitative Results and Patient Diagnostic Histories

Table 1 shows the descriptive data for the sample. Two issues regarding the data require clarification: firstly, despite NPR recording that every patient had a diagnosis of CDD, parents reported only 75% of the sample had a current diagnosis of CDD (17% had a diagnosis of PDDNOS and ASD, and 8% had a diagnosis of ASD). Additionally, 100% of parents reported a clear regression of abilities in their child (meeting a key diagnostic criterion of CDD) and treatment service personnel had ascertained that the patient met CDD diagnosis criteria before forwarding the study invitation to the family. Due to the similarities in symptoms, and the lack of clarity in CDD differentiation, it was decided to include these cases in the study. Unfortunately, due to the restrictions of the recruitment method, further assessment (for example via a review of patient journals) as to why these cases were not currently diagnosed with CDD, was not possible. Secondly, one case was reported with regression before the age of 2 years (i.e. earlier than in standard CDD criteria). This case *was* reported by the parent to have a confirmed CDD diagnosis given by treating medical personnel.

**Table 1:** Descriptive patient data

Variable	Percentage of sample (n = 12)
Current diagnosis (parental report)^a^	
CDD	75 (n = 9)
PDDNOS & ASD	17 (2)
ASD	8 (1)
Current age	
3–10 years	33 (4)
10–18 years	50 (6)
18 + years	17 (2)
Gender	
Male	92 (11)
Female	8 (1)
Care situation	
Living at home full-time	33 (4)
Living at home, with respite in a care home	25 (3)
Living in care full-time	42 (5)
Evident regression in child’s abilities?	
Yes	100 (12)
No	0
Age of regression onset^b^	
Before 2 years	8 (1)
2–4 years	67 (8)
4–7 years	8 (1)
7 + years	8 (1)
Regression symptoms	
Loss/Decline of language skills	100 (12)
Loss/Decline of social skills	83 (10) (17% report this may already have been present)
Loss/Decline of motor skills	50 (6)
Loss/Decline of toilet training abilities	50 (6)
Hallucinations	33 (4)
Fear/awareness ‘something was wrong’	33 (4) (an additional 8% report crying/sadness)
Developing repetitive behaviours	83 (10)
Epilepsy (*not included in questionnaire*)	25 (3) (an additional 17% with sub-clinical ‘overactivity’ or seizures)

^a^100% of sample at some point registered with CDD according to NPR

^b^8% Unsure

Further to the issue of diagnosing CDD in patients, parents were asked to report as fully as they could the assessments their child had gone through before receiving the CDD diagnosis. A huge variety of tests were described, including: assessment for metabolic disorders, brain tumours, and Landau-Kleffner syndrome; tests for genetic abnormalities (and in some cases gene testing of the parents themselves); MR scans and EEG assessments (commonly reported); tests of motoric abilities and physiotherapeutic assessments; various blood tests; psychiatric assessments of both verbal and non-verbal communication; and hearing and sight tests (commonly reported in early phases of diagnosis). Outside of public health services, families had also privately sought hair mineral analysis and chelation therapy. Many parents reported that they felt CDD had been diagnosed as a ‘last resort’ when no other underlying cause had been discovered for the child’s regression. It was clear from parent responses that many had read extensively on potential causes for the regression and several mentioned being ‘desperate’ to find an answer to what had happened to their child. Several parents also mentioned that they had themselves suggested potential causes and assessments to treatment service personnel: in some cases, parents said they had initiated or encouraged some of the avenues of testing themselves.

In addition to this information, parents were asked to report on previous diagnoses their child had received and any current additional diagnoses. Two parents reported that a child now diagnosed with CDD had previously been diagnosed with autism before being diagnosed with CDD (one of these had received the CDD diagnosis in adulthood); one parent reported their child had briefly been diagnosed with Asperger’s syndrome, then PDDNOS, before being diagnosed with CDD; and one parent reported that their child had previously been diagnosed with schizophrenia, and that pantothenate kinase-associated neurodegeneration had been considered likely (then removed as a diagnosis), before as an adult being diagnosed with CDD. Forty-two percent of parents reported their child having a separate diagnosis of intellectual disability (this is likely to be underreported from the context of other patient behavioural descriptions which suggested most patients having some intellectual disability). One patient with CDD also had a diagnosis of Tourette’s syndrome and obsessive–compulsive disorder, and one patient with CDD also had a diagnosis of Cowden syndrome. Additionally, 25% of the sample were reported to have epilepsy (see Table 1).

Parents were also asked to report whether they themselves or any other members of their immediate or extended family had any diagnosed psychological problems or learning difficulties. In almost all cases no such conditions were reported. One parent responded that the father of the CDD patient possibly had (undiagnosed) ADHD. One parent reported that a sibling of the CDD patient had dyslexia. In the extended family, one parent reported a relative with ADHD, one reported a relative with schizophrenia, and one reported a relative with diagnosed Asperger’s syndrome (two parents also reported that a family member had traits of Asperger’s syndrome, but these were undiagnosed).

Table 2 shows the participants responses to the questions employing a 1–7 scale assessing the effects of the child’s regression on parents and families. Of particular note is the high percentage of participants who responded with the maximum value of 7 on all three measures.

**Table 2:** Scale question responses

Question	Participant responses (%) n = 12						
	1 (Not at all)	2	3	4	5	6	7 (Very much)
Did your child developing difficulties make family life difficult?	8	0	0	17	17	8	50
Did your child developing difficulties cause you and your family stress and anxiety?	0	0	0	0	25	0	75
Was your child developing difficulties traumatic for you and your family?	0	17	8	8	8	0	58

### Qualitative Results

The study also asked several ‘open’ questions regarding the regression of the CDD patient and how parents and their family had experienced the patient’s regression. The thematic analysis produced a topic which specifically covered these elements regarding regression under the title ‘I don’t understand that a personality can change so much’. This theme is presented below.^1^

‘I don’t understand that a personality can change so much’.

One of the key themes to emerge regarding regression was the effects on parents of the changes in the child. All parents had experienced regression in their child’s abilities and the entire patient sample showed deficits in language and social skills. The majority of parents reported that regression happened gradually, over a period of ≥ 6 months to over a year, with a minority reporting a more abrupt decline over a period of a few months. Language and social skills declining were usually the first signs parents noticed of regression. K. describes her child’s decline in language:

It used to be that if the doorbell rang s/he said ‘‘maybe E. is here!’’… a mate had come to visit… and then s/he didn’t pay attention to noises anymore, that was a bit of the first I thought was strange… and then from him/her saying ‘‘mummy I want biscuits’’… and so suddenly s/he said ‘‘mummy want isca’’… and then it went to being just ‘‘isca’’… and then to that s/he just stood and pointed… It happened over nearly 8, 9 months from when s/he started to muddle speech until it disappeared completely… ‘‘yes’’ and ‘‘no’’, they went right at the end… and after that s/he hasn’t said anything.

It was also common for parents to report that the beginning of regression was accompanied by the child having sleep-disturbances, as T. recounts:

S/he was about 3 years old… we noticed gradually that words disappeared, and also that s/he started to have a lot of trouble sleeping—many nights a week s/he woke around 1–2am and was awake for several hours before s/he got to sleep again in the morning. At that time s/he went from being able to say simple sentences (‘sit sofa read book’)… name foods and things s/he wanted… use set-phrases like ‘happy birthday’, ‘thanks for dinner’… to not using words at all… At first I was irritated, before I understood that s/he couldn’t ‘find’ the word. From about the age of 5 s/he hasn’t used words at all, but s/he understands a lot of what we say to him/her.

Several parents reported that when regression was gradual, they had not immediately noticed the decline. The slow onset in these cases led to one parent saying they had ‘*waited for longer than they should have*’ before seeking help. Several parents said they had initially felt ‘relaxed’, that things would ‘be fine’, or that ‘*all children develop differently*’. Some expressed retrospectively that they ‘*didn’t take it on-board*’. Some reported that their initial concerns about their child were not taken seriously by school and health personnel. Once the child’s problems had become more evident, parents described myriad emotional responses to the regression, including worry, sadness, stress, anxiety, and shock. W. called their child’s regression ‘*the worst thing that’s happened to us*’.

In some cases, parents reported that in contrast to a gradual decline in skills, there was a dramatic period of hallucinations and ‘psychosis’, accompanied with challenging behaviour, at the start of the child’s regression. O. recounts:

S/he started to be scared of everything. S/he looked at the patterns on the walls… and would say that snakes were… attacking his/her (sibling). And that people were coming to catch us…s/he became scared of: carpets, shadows, walls, trees, and almost everything around him/her…and after that s/he cried a lot and started to bite, hit and scratch him/herself and others… Sometimes s/he sat on the sofa… and stared into a corner and said ‘‘H. is coming… she’s gone’’. We don’t know if s/he was hallucinating or what s/he meant by that. After 3–4 months s/he cried less, it was then that I noticed s/he wasn’t speaking normally, and if I said something s/he would run round and round and repeat what I had said… S/he used to turn off the lights in the room we were sitting in and come over to us, very near my face, or his/her father’s, and in a way try to identify us…

In some cases, this period of decline also saw the child become incontinent and/or lose motor skills, as F. reports:

‘…became incontinent, massive functional decline which can easily be seen in the video-documentation, regression in drawings (had drawn with detail, they became just lines)…’

Unsurprisingly, parents in these situations reported feelings of shock and confusion, and all reported the regression itself as a period of high stress and trauma. Several parents expressed that seeing their child regress left them comparing ‘before’ and ‘after’, and this caused trouble ‘reconciling’ with their child’s identity, as W. describes:

I don’t really understand it… how can an averagely intelligent person stop being averagely intelligent?… and have a learning disability… I don’t understand how a personality can change so much… I think it was a couple of years to make R. (child) into one person… there was R. before, and there was R. after… s/he wasn’t an individual, in a way.

Further, parents reported that the profound level of change, and having seen decline in abilities, left them hoping to see those abilities return, as K. says, ‘*…that is sort of what we have always thought and hoped for the whole time…that in a way they* [abilities] *might still be there somewhere and they can come back again after a while…*’.

## Discussion

### Symptomatology

There are very few published studies from anywhere in the world that collect information on CDD symptomatology, and none that have previously documented cases in Norway. To this end the documenting of the symptomatology of 12 CDD patients in Norway may in itself be of some value to the further study of the condition.

There were discrepancies between Norwegian Patient Registry (NPR) information (and treatment service personnel’s validation of symptoms) indicating CDD in patients, and parental reports of these patients not having a CDD diagnoses. This occurred in 25% of cases in the study. This may be illustrative of the lack of clarity surrounding differentiation of CDD as a diagnosis (Kurita et al., 2005; Volkmar & Rutter, 1994), or the usage of differing terminology for the diagnosis within healthcare and treatment services. Future research should endeavour to recruit larger samples of patients with unexplained regression (across CDD, ASD, and PDDNOS patients) to ascertain more clearly the similarities or differences that may constitute variant diagnoses.

Of the sample (n = 12), 92% were male, which is higher than the 5.3:1 male: female ratio reported in Mehra et al. (2018) (though our sample size was small). Age of regression was consistent with previous studies (Mehra et al., 2018; Volkmar, 1992): a significant majority where onset was between the ages of 2 and 4 years (67%), one case with onset between 5 and 6 years, and one after the age of 7 years. One case was reported with regression before the age of 2 years (this case was reported by the parent as having a confirmed CDD diagnosis). Considering that the majority of the patient sample were under 18 years old (83% ≤ 18 years), a large percentage were in permanent care (42%), which reflects the extent of challenging behaviours these patients displayed, supporting the finding of Malhotra and Gupta (2002) that such behaviour is common in CDD. However, the extent of challenging behaviour reported varied significantly (with a minority of cases showing little or no challenging behaviour), indicating these challenges are not synonymous with CDD.

Symptoms of regression reported by parents in the sample were consistent with those reported in the review by Mehra et al. (2018). Most notably: 100% of patients had shown a decline in language skills; 100% had deficits in social skills (though 17% may have had deficits pre-regression); and 83% had developed repetitive behaviours. The high prevalence of the loss of bowel and bladder control (50%), also supports previous findings that this is more common in CDD than ASD (Mehra et al., 2018). A majority of parents indicated that regression had happened gradually over a period of ≥ 6 months to a year, whilst a minority reported a more rapid onset of symptoms in a period of a few months. Future research should concentrate on the chronology and timespan of regression symptom onset to better ascertain whether these factors indicate potential markers for differential diagnostics.

A significant minority of patients were reported to have had hallucinations during regression (33%, n = 4) and a further 33% showed signs of fear, or ‘awareness that something was wrong’. There was significant overlap between these two groups: 3 patients were reported to have both shown awareness something was wrong/fear *and* experienced hallucinations. One patient was reported as having shown fear/awareness something was wrong, but without signs of experiencing hallucinations; a further individual patient was reported by parents to have experienced hallucinations, but without showing awareness something was wrong/being fearful. Additionally, one patient was reported to have shown a large amount of sadness and crying during symptom onset (this patient did not present with hallucinations or other signs of fear/awareness ‘something was wrong’). The presence of these symptoms in a minority of patients supports previous findings (Kurita, Koyama, et al., 2004; Westphal et al., 2013) and these could conceivably represent patient sub-groups (the study found no other obvious similarities within these groups).

Of the patients in the study, 25% had epilepsy. A further 17% were reported with signs of overactivity on EEG readings, or as having experienced seizures, but these were subclinical and not diagnosed as epilepsy (these figures may be underreported as the questionnaire did not explicitly list epilepsy on the patient symptom measure). This supports previous findings that whilst epilepsy is prevalent amongst CDD patients, it is not synonymous with the condition (Mehra et al., 2018). Further work on the presence of epilepsy in CDD patients (as well as potential links between CDD and conditions such as Landau-Kleffner syndrome, Stefanatos et al., 2002) and on the presence of hallucinations and fear in CDD patients at onset, could be useful in ascertaining whether these factors indicate clinically differing subgroups within CDD (or differing diagnoses altogether).

Whilst this picture of CDD patient symptomatology is based on parental report, rather than clinical interview, this still provides an important description of the sample (due to the rarity of CDD, identifying patients and unifying reports from hospitals is also extremely challenging). The high prevalence of domain-general regression in language and social skills, development of repetitive behaviours, and the late onset age of regression, support previous findings that these may be differential characteristics of CDD as a distinct diagnosis (Mehra et al., 2018).

Parent reports on the presence of psychological disorders or learning disabilities within the family showed little indication of potentially associated conditions (no parents reported any confirmed psychiatric diagnoses of any kind in themselves or their partner; no siblings with autism or significant learning disabilities were reported; one case of ADHD, one of diagnosed Asperger’s syndrome, and one of schizophrenia in extended families were reported). Whilst caution is of course advised in drawing any conclusions from such a small sample, there was little obvious indication of intra-familial correlation between CDD and other ASD conditions, learning disabilities, or any other psychological diagnoses, in the study. Assessing larger samples of patients with CDD (and ‘regressive autism’), and their families, for potential links to other conditions would potentially assist research on establishing whether these cases are linked to autism more generally or are aetiologically independent (Gupta et al., 2017).

Most patients had been through an extensive battery of tests and assessments before a diagnosis of CDD had been made. In both cases where the CDD patient was now an adult, this diagnosis had been made in adulthood (in one case 26 years after regression occurred). The variety of tests employed and length of assessment before obtaining a diagnosis is likely to be an indication of the uncertainty surrounding CDD as a diagnosis and indicative of the sense several parents reported that CDD was a diagnosis of ‘last resort’. Similar prolonged gaps between initial assessment and diagnosis of patients were reported in a study of Fragile X syndrome (a condition more common than CDD) by Gabis et al. (2017). These prolonged experiences of testing were evidently something parents had found draining and emotional and further research should focus on assessing how such experiences might affect both patient and family wellbeing.

### Regression Effects on Parents

The regression of the child was a factor that was associated with a variety of psychological effects which may be specific to the CDD parent group. Whilst this study was unable to quantify effects on parents from the child’s regression through specific clinical assessments of stress and anxiety in parents (or other psychological effects, such as depression), the high self-reporting of significant levels of stress, anxiety, and trauma (see Table 2) are indicators of these factors being important in the wellbeing of parents of CDD patients. Such effects are unsurprising but, to the best of the authors’ knowledge, there has been no previous research specifically focusing on the effects of CDD on patients’ families.

A limited number of studies on Rett syndrome (a PDD with regressive elements) have focused on family functioning in the long-term and found links to increased anxiety and stress (Cianfaglione et al., 2015), depression (Sarajlija et al., 2013), and lower marital satisfaction (Perry et al., 1992), with stress, anxiety, and depression amongst mothers stable over a 16-month period (Cianfaglione et al., 2016). The specific effects of the regression experience itself in Rett syndrome are again however, little studied. The present study’s results indicate that the observed psychological effects seen in families of Rett syndrome patients are likely to be relevant in families of CDD patients. The study also underlines that the regression phase itself should be seen as an independent factor in the complex picture of how parents and families are affected by CDD. Future research should focus on assessing the effects of regression directly through clinical assessment, both at the time of patient regression, and in the long-term.

Some parents had been additionally affected by seeing their child go through periods with hallucinations, or by seeing them verbally expressing their fear and suffering during regression. Further research should focus on whether CDD patients with symptoms of hallucinations or awareness of regression onset could constitute distinct patient sub-groups (Agarwal et al., 2005; Kurita, Koyama, et al., 2004; Westphal et al., 2013). Additionally, these factors should be considered in terms of their potential impact on parents, family, and other persons close to the patient.

The lack of knowledge on CDD may not only create challenges for clinicians and hinder diagnostic differentiation of CDD but may also affect parents. The study revealed that parents often felt CDD was a diagnosis of ‘last resort’. Together with the fact that the causes of CDD remain unknown, this can be considered an extra emotional burden on parents, who are left wondering ‘why this has happened’. Previous studies of regression in patients with autism (Davidovitch et al., 2000; Goin-Kochel & Myers, 2005) have had similar findings, with parents reporting a desire for ‘an explanation’. It is also potentially of consideration that failing to specifically address the effects of such unexplained regression on patient’s families may negatively impact the provisioning of appropriate support.

Furthermore, unexplained regression creates a sense of uncertainty as to whether the child’s abilities might return. Cases of limited regaining of language skills in CDD do exist in the literature (Mordekar et al., 2009). Although some research suggests that both in ASD regression and CDD, later regression and more domain-general skill losses are related to lower rates of regaining language skills, there is little knowledge on how common regaining speech is (Boterberg et al., 2019; Matson & Mahan, 2009). The desire for regaining language typifies what several parents reported feeling about the regression of their child: a sense of ‘loss’ due to the changes the child went through, which in some cases led to comparison of the ‘two identities’ their child had had, and difficulty reconciling to this. This sense of ‘loss’ corresponds to the findings of Kirk et al. (2014) in parents of children with a TBI (traumatic brain injury).

The lack of concrete information regarding CDD as a condition, and specifically the uncertainty surrounding long-term prognosis for affected patients regarding regaining ‘lost’ abilities, is likely to compound the uncertainty and emotional burden parents experience.

### Limitations

There are a couple of limitations to consider with this study. Firstly, the recruitment method for the study relied on sending letters via contacts at treatment services. Whilst this maintained anonymity for families, it also meant that validation of patient details was carried out by 20 individuals and it was impossible to verify these checks were carried out to the same standard. Discrepancies in these standards could conceivably also have impacted the response rate negatively. The response rate was somewhat lower than other health psychology studies of non-patients (Robb et al., 2017). The length of the questionnaire may have also potentially impacted the response rate (although Robb et al., 2017, found this had little effect) whilst conversely enabling gathering detailed and varied responses.

As discussed, there were discrepancies between NPR data and parental reports of patient diagnoses. To better ascertain the reasons for these discrepancies, it would have been optimal to have clinical specialists conduct a journal review of the entire sample. Additionally, participants responded by handwritten or online questionnaire, or were interviewed in person or by phone. These varying methods of participation may have influenced the way respondents answered. However, offering several methods of participation may also have increased the response rate (due to individual preferences for responding verbally or in writing). Differing participation methods can also be argued to be a form of ‘methodological triangulation’ which can improve data reliability (Farmer et al., 2006).

## Conclusion

The study supported existing CDD diagnostic criteria with universal regression of language and social skills in the sample and onset in the majority of cases between the ages of 2–4 years. Regression was linked to significant effects on parental wellbeing in terms of reported stress and trauma, as well as specific feelings of ‘loss’. There were also indications that lengthy patient assessments, the usage of CDD as a diagnosis of ‘last resort’, and uncertainty about CDD patient prognoses, were additional factors negatively affecting parental wellbeing.

## Footnotes

1 To protect anonymity all parent names are replaced by a randomly chosen letter. Due to the gender imbalance in the sample patient gender has also been anonymised and is replaced with the pronoun s/he.
